# Application of the Brazilian patch test panel in the diagnosis of allergic contact dermatitis to cosmetics^☆^

**DOI:** 10.1016/j.abd.2021.06.009

**Published:** 2022-06-25

**Authors:** Ana Luiza Castro Fernandes Villarinho, Maria das Graças Mota Melo, Liliane Reis Teixeira

**Affiliations:** Department of Work-related Dermatology, Centro de Estudos da Saúde do Trabalhador e Ecologia Humana, Escola Nacional de Saúde Pública Sergio Arouca, Fundação Oswaldo Cruz, Rio de Janeiro, RJ, Brazil

Dear Editor,

Cosmetics are products that are widely used by all age groups, making adverse reactions a public health problem. The most common adverse event is irritant contact dermatitis, but allergic contact dermatitis (ACD) often receives more attention, as it is related to more severe reactions.^1^ Cosmetic constituents, such as fragrances and preservatives, account for a good number of ACD cases and the diagnosis is made through clinical history and physical examination, which is corroborated by the patch test (PT).^2^, ^3^ In Brazil, the standard panel includes 30 substances, of which at least 18 elements are found in cosmetics, in addition to the cosmetic panel with ten additional allergens. The performance of the PT can be increased with the use of natural cosmetics, especially when there is difficulty in having access to different panels and individual allergens. The present study evaluated the prevalence of allergy to cosmetics, the involved allergens, characteristics of the affected population, and the performance of the test with natural cosmetics.

A cross-sectional study was conducted based on PTs applied between 2013 and 2017 at a Work-Related Dermatology Service and at an Allergic Dermatosis Outpatient Clinic in the city of Rio de Janeiro. All patients underwent the PT with the standard and Brazilian cosmetic panels (FDA Allergenic and Asac Pharma). In those with suspected ACD induced by personal-use cosmetics, if the product could not be rinsed off, it was tested *in natura*. It is noteworthy that nail polish was applied directly to the patch test strips and only after drying, the tapes were applied on the back of the patients. Readings were taken after 48 and 96 h.

Of the 768 tests performed, 251 (32.7%) were diagnosed as ACD to cosmetics, predominantly women (201/80.1%) with a mean age of 43.7 years (SD = 14.7). Overall, the hands were the site most frequently affected by ACD to cosmetics (123/49%), with eczema on the palms observed in 31.5% (79) of the cases and on the back of the hands in 29.5% (74). Compared with the males, females had a higher prevalence of lesions on the face (p < 0.02) and on the lower limbs (p < 0.03; Table 1).

**Table 1:** Demographic and clinical characteristics of patients with allergic contact dermatitis to cosmetics, according to the MOAHLFA index.

		Sex		p-Value
	Total	Male	Female	
	n	n (%)	n (%)	
**M**(ale) (sex)	251	50 (19.9)	201 (80.1)	‒
**O**(occupational)	75	12 (16)	63 (84)	0.16
**A**(topic dermatitis)	16	4 (25)	12 (75)	0.75^a^
**H**(and)	123	22 (17.9)	101 (82.1)	0.43
**L**(eg)	97	26 (26.8)	71 (73.2)	<0.03
**F**(ace)	91	11 (12)	80 (88)	<0.02
**A**(ge) > 40 years	148	33 (22.3)	115 (77.7)	0.26

a Fisher’s test.

The analysis of positive PT results showed that the Kathon CG preservative (150/60.2%) was the most prevalent allergen in the standard panel among patients with ACD to cosmetics, followed by nickel sulfate (91/36.5%), perfume MIX (47/18.9%), thimerosal (47/18.9%), paraphenylenediamine (31/12.4%), cobalt chloride (31/12.4%) and formaldehyde (25/10%). In the cosmetics panel, the substances with the higher prevalence of positive results were Tosylamide/formaldehyde resin (35/14.1%), triethanolamine (24/9.6%), Bronopol (8/3.2%), Germall 115 (8/3.2%) and Amerchol L-101 (7/2.8%). It is noteworthy, however, that among the most prevalent allergens in the standard panel, the only ones that showed current clinical relevance greater than 50% were Kathon CG (99%), perfume MIX (87.5%), paraphenylenediamine (68.8%) and formaldehyde (68%), while all the most prevalent substances in the cosmetics panel showed significant relevance (Table 2).

**Table 2:** Prevalence of positive patch tests and current clinical relevance of cosmetic allergens present in the standard panel and the constituent elements of the cosmetics panel.

Standard panel	Positive tests	Current clinical relevance
	n (%)	n (%)^a^
**Fragrances**		
Balsam of Peru	15 (6)	12 (80)
Perfume – MIX	48 (19.1)	42 (87.5)
**Preservatives**		
Para-tertiary butylphenol	1 (0.4)	0
Formaldehyde	25 (10)	17 (68)
Irgasan DP300	2 (0.8)	0
Kathon CG	150 (60.2)	149 (99)
Paraben MIX	4 (1.6)	2 (50)
Propylene glycol	3 (1.2)	3 (100)
Quaternium-15	7 (2.8)	3 (42.9)
Thimerosal	48 (19.1)	0
**Antioxidants**		
Hydroquinone	6 (2.4)	0
**Emulsifiers**		
Lanoline	5 (2)	5 (100)
**Hair dyes/other hair cosmetics**		
Paraphenylenediamine	31 (12.4)	22 (68.8)
PPD	13 (5.2)	1 (7.7)
**Nail cosmetics**		
Colophony	10 (4)	2 (20)
**Metals**		
Potassium bichromate	17 (6.8)	6 (35.3)
Cobalt chloride	32 (12.7)	13 (40.6)
Nickel sulfate	92 (36.7)	38 (41.3)
**Cosmetics series**		
**Preservatives**		
Sorbic acid	0	0
Bronopol	8 (3.2)	5 (62.5)
Chloroacetamide	1 (0.4)	0
Chlorhexidine	0	0
GERMALL 115 (Imidazolidinyl urea)	8 (3.2)	5 (62.5)
**Antioxidants**		
BHT	1 (0.4)	1 (100)
**Emulsifiers**		
Amerchol l-101	7 (2.8)	4 (66.7)
Triethanolamine	24(9.6)	18 (75)
**Hair dyes/other hair cosmetics**		
Ammonium thioglycolate	1 (0.4)	1 (100)
**Nail cosmetics**		
Tosylamide/Formaldehyde Resin	35 (14)	29 (82.9)

a Valid percentage in relation to positive tests, considering the inconclusive ones.

In 93 cases (37.1%) the test was carried out with natural cosmetics, and the best performance (positive tests/total number of tests) was obtained with nail polishes (61.8%), facial/body moisturizer (53.8%), sunscreen (41.7%) and perfumes/cologne (36%; Table 3). It is noteworthy that among patients who were tested with natural nail polishes and had a positive result (21), eight were negative for Tosylamide/formaldehyde resin and did not have relevant positive tests for other allergens in the standard panel. In these cases, if the nail polish had not been tested, it would not have been possible to define the ACD agent.

**Table 3:** Performance of patch tests carried out with natural cosmetics.

Type of cosmetic	Total of tests	Positive results	Performance
	n	n	%
Face/body moisturizer	39	21	53.8
Nail polish	34	21	61.8
Perfumes/cologne	25	9	36
Hair styling cream	20	2	10
Deodorant	17	2	11.8
Sunscreen	12	5	41.7
Lipstick/lip moisturizer	8	2	25
Blush	4	1	25
Foundation	7	2	28.6
Eye pencil/ eyeliner	4	1	25

Again, Kathon CG was the allergen most significantly associated with the involvement of specific body segments (Table 4). Knowledge of the association of allergens with the most affected sites guides the screening for possible cosmetics involved in the condition and helps restrict products before the PT is applied.

**Table 4:** Association between lesion site and the most prevalent allergens by body segment of the Brazilian standard and cosmetic panels that showed current clinical relevance.

Site	Allergens	Positive tests	p-Value
		n (%)	
Scalp	Kathon CG	9 (56.25)	0.53
	Nickel sulfate	5 (31.25)	0.63
	Paraphenylenediamine	6 (37.5)	<0.01
Face	Kathon CG	46 (53.5)	<0.01
	Nickel sulfate	35 (40.7)	0.35
	Tosylamide/Formaldehyde Resin	19 (22.1)	<0.02
Eyelids/periorbital	Kathon CG	10 (31.25)	<0.01
	Nickel sulfate	15 (46.9)	0.21
	Tosylamide/Formaldehyde Resin	11 (34.4)	<0.01
Lips/perioral	Kathon CG	9 (47.4)	0.13
	Nickel sulfate	10 (52.6)	0.14
	Thimerosal	4 (21.1)	0.82
	Tosylamide/Formaldehyde Resin	4 (21.1)	0.43
Cervical/inframentonian region	Kathon CG	26 (53)	0.09
	Perfume MIX	13 (26.5)	0.19
	Nickel sulfate	23 (46.9)	0.10
Trunk	Kathon CG	68 (76.4)	<0.01
	Perfume MIX	19 (21.3)	0.67
	Nickel sulfate	30 (33.7)	0.43
Axillae	Perfume-MIX	7 (43.7)	<0.01
	Kathon CG	11 (68.7)	0.65
	Nickel sulfate	6 (37.5)	0.96
Upper limbs	Kathon CG	92 (76.7)	<0.01
	Perfume-MIX	30 (25)	<0.05
	Nickel sulfate	40 (33.3)	0.25
Hands	Thimerosal	28 (23.9)	0.06
	Kathon CG	88 (75.2)	<0.01
	Nickel sulfate	44 (37.6)	0.81
Lower limbs	Kathon CG	73 (80.2)	<0.01
	Perfume MIX	20 (22.7)	0.53
	Nickel sulfate	29 (31.9)	0.21
Feet	Kathon CG	46 (78)	<0.01
	Thimerosal	12 (20.3)	0.77
	Nickel sulfate	16 (27)	0.07

Kathon CG is a preservative that consists of a mixture of methylchloroisothiazolinone and methylisothiazolinone (MI), at a 3:1 ratio, respectively, found in cosmetics, industrial and cleaning products, and paints. The high prevalence of positive tests for this mixture, reaching 60% of cases of ACD to cosmetics, possibly reflects the occurrence in our country of the MI portion sensitization epidemic, which has been reported worldwide since 2010. ^4^ Despite this, MI alone has not yet been added to the national standard panel.^5^ Metals can be found in eye shadow (chrome and nickel), mascara (chrome), hair dye (cobalt and nickel), and nail polish (cobalt), among others. Although the positive patch testing with nickel sulfate is prevalent, its clinical and cosmetic relevance to ACD is often difficult to establish.^6^ The screening of ACD to fragrances with the standard panel is carried out using Perfume mix and Balsam of Peru. These substances are also present in cleaning products, fabrics, and condiments, among others, increasing the possible sources of exposure. In the present study, the prevalence of positive tests for the Perfume mix was 19.1%; while the world average of positive tests for this mixture ranges from 4% to 11%.^7^ The higher prevalence observed can be explained by the fact that the analysed sample consisted only of patients known to have ACD from cosmetics.

Regarding Thimerosal, despite the high prevalence of positive tests, none of them showed clinical relevance. For this reason, this allergen has already been excluded from the standard North American and European panel. Paraphenylenediamine is added to hair dyes to intensify the color and increase dye durability, which ultimately explains its association with scalp lesions.^8^ Sensitization to formaldehyde and its releasers, such as Quaternium 15, Bronopol, and Germall-115, occurs either alone or in combination. In the present study, four individuals allergic to formaldehyde also showed sensitization to at least one of its releasers. In Brazil, formaldehyde is tolerated as a cosmetic preservative and nail hardener, at maximum concentrations of 0.2% and 5% respectively.^9^

The routine performance of the cosmetic panel is recommended only when ACD to this type of product is suspected, aiming to increase the accuracy of the patch test. However, in the present study, both panels were tested consecutively because the national panel has not been updated for some years and the objective was to increase the test performance. The European Contact Dermatitis Society recommends that an allergen should be included in a country standard panel when sensitization to it exceeds 0.5–1% of the performed tests and is clinically relevant.^10^ Therefore, the addition of some elements of the cosmetic panel to the standard Brazilian panel could be considered for regular testing. It is noteworthy, however, that it would be necessary to assess whether the sample analysed in this study is representative of the Brazilian population. Additionally, other preservatives used in cosmetics and with a high prevalence of sensitization in other countries, such as methylisothiazolinone, methyldibromo glutaronitrile, and cocamidopropyl betaine could be considered.

## Financial support

None declared.

## Authors' contributions

Ana Luiza Castro Fernandes Villarinho: Statistical analysis; design and planning of the study; drafting and editing of the manuscript; collection, analysis, and interpretation of data; intellectual participation in the propaedeutic and/or therapeutic conduct of the studied cases; critical review of the literature.

Maria das Graças Mota Melo: Drafting and editing of the manuscript; effective participation in research orientation; intellectual participation in the propaedeutic and/or therapeutic conduct of the studied cases; critical review of the literature; critical review of the manuscript.

Liliane Reis Teixeira: Statistical analysis; approval of the final version of the manuscript; effective participation in research orientation; critical review of the manuscript.

## Conflicts of interest

None declared.
